# Clinical Relevance of Human Immunodeficiency Virus Low-level Viremia in the Dolutegravir era: Data From the Viral Load Cohort North-East Lesotho (VICONEL)

**DOI:** 10.1093/ofid/ofae013

**Published:** 2024-01-08

**Authors:** Maurus Kohler, Jennifer A Brown, Nadine Tschumi, Malebanye Lerotholi, Lipontso Motaboli, Moliehi Mokete, Frédérique Chammartin, Niklaus D Labhardt

**Affiliations:** Division of Clinical Epidemiology, Department of Clinical Research, University Hospital Basel, Basel, Switzerland; Division of Clinical Epidemiology, Department of Clinical Research, University Hospital Basel, Basel, Switzerland; Division of Clinical Epidemiology, Department of Clinical Research, University Hospital Basel, Basel, Switzerland; Division of Clinical Epidemiology, Department of Clinical Research, University Hospital Basel, Basel, Switzerland; Ministry of Health Lesotho, Maseru, Lesotho; SolidarMed, Partnerships for Health, Maseru, Lesotho; SolidarMed, Partnerships for Health, Maseru, Lesotho; Division of Clinical Epidemiology, Department of Clinical Research, University Hospital Basel, Basel, Switzerland; Division of Clinical Epidemiology, Department of Clinical Research, University Hospital Basel, Basel, Switzerland; Division of Infectious Diseases and Hospital Epidemiology, University Hospital Basel, Basel, Switzerland

**Keywords:** antiretroviral therapy, dolutegravir, HIV, low-level viremia, virological failure

## Abstract

**Background:**

Human immunodeficiency virus low-level viremia (LLV) is associated with subsequent treatment failure at least with non nucleoside reverse transcriptase inhibitor (NNRTI)-containing antiretroviral therapy. Data on implications of LLV occurring under dolutegravir, which has largely replaced NNRTIs in Africa, are scarce, however.

**Methods:**

We included adults with human immunodeficiency virus in Lesotho who had ≥2 viral loads (VLs) taken after ≥6 months of NNRTI- or dolutegravir-based antiretroviral therapy. Within VL pairs, we assessed the association of viral suppression (<50** **copies/mL) and low- and high-range LLV (50–199 and 200–999** **copies/mL, respectively) with virological failure (≥1000** **copies/mL) using a mixed-effects regression model. Participants could contribute VLs to the NNRTI and the dolutegravir group.

**Results:**

Among 18 550 participants, 12 216 (65.9%) were female and median age at first VL included was 41.2 years (interquartile range, 33.4–51.5). In both groups, compared with a suppressed VL, odds of subsequent virological failure were higher for low-range LLV (NNRTI: adjusted odds ratio; 95% confidence interval: 1.9; 1.4–2.4 and dolutegravir: 2.1; 1.3–3.6) and high-range LLV (adjusted odds ratio; 95% confidence interval, 4.2; 3.1–5.7 and 4.4; 2.4–7.9).

**Conclusions:**

In the dolutegravir era, LLV remains associated with virological failure, endorsing the need for close clinical and laboratory monitoring of those with a VL ≥50 copies/mL.

Since 2021, the World Health Organization (WHO) has recommended a regimen switch for people with human immunodeficiency virus (HIV) and taking antiretroviral therapy (ART) who had 2 consecutive viral loads (VLs) above 50 copies/mL with the second above 1000 copies/mL [1]. This reflects a substantial change to previous WHO guidelines in which any VL <1000 copies/mL was considered to be “suppressed” and thus 2 consecutive VLs ≥1000 mL were required a switch to a second-line regimen [2]. The WHO lowered the VL threshold based on accumulated observational evidence that low-level viremia (LLV) is associated with resistance mutations [3–5], subsequent virological failure [6–11], and acquired immunodeficiency syndrome (AIDS)-defining events and mortality [12, 13]. Furthermore, 1 open label randomized controlled trial showed that a switch to second-line regimen in those with persistent LLV led to a higher rate of viral resuppression [14].

However, evidence on the association of LLV with virological failure mainly derives from studies of those taking nonnucleoside reverse transcriptase inhibitors (NNRTIs) as the core agent of their ART regimen [6–11]. Today, all major guidelines, including the WHO guidelines, recommend integrase strand transfer inhibitors (INSTIs) as the core agent for first-line ART [1, 15–17], and dolutegravir, a second-generation INSTI, has become the ART core agent for most people with HIV in Africa [18]. It is, however, not clear if findings on LLV and their clinical implications in people taking NNRTI-based ART can be transferred to other drug classes, such as dolutegravir [1, 15–17]. The WHO guidelines state that evidence on LLV occurring in people taking INSTI-based or protease inhibitor–based as opposed to NNRTI-based ART is very limited [1].

In this study, we assess and compare the association of LLV with virological failure among adults with HIV taking dolutegravir- and/or NNRTI-based ART in the prospective open Viral Load Cohort North-East Lesotho (VICONEL).

## METHODS

### Study Design and Setting

VICONEL is a prospective open cohort of people with HIV established in Lesotho, southern Africa, in December 2015. VICONEL includes VL test results from 3 hospitals, 18 health centers, and 2 private clinics in rural districts of Butha-Buthe and Mokhotlong. As part of routine care, blood samples for VL measurement are taken at the participating healthcare facilities per the schedule of the Lesotho National Guidelines. Samples are processed at Butha-Buthe Government Hospital on the Cobas 4800 platform (F. Hoffman-La Roche AG, Basel, Switzerland) with a lower detection limit of 20 copies/mL or, for a small subset, with point-of-care instruments (GeneXpert, Cepheid, Sunnyvale, CA, USA) with a lower detection limit of 40 copies/mL at 3 other participating healthcare facilities. VL results and associated demographic and treatment metadata are uploaded to the secured VICONEL database. Further details on the setup and design of VICONEL have been reported previously [19]. The Lesotho national rollout of dolutegravir replacing NNRTI as the ART core agent for first-line regimens started in 2019, with most people with HIV in Lesotho transitioned in 2020.

### Data

We included adults (aged ≥18 years) with HIV with at least 2 VLs taken during NNRTI-based and/or at least 2 VLs taken during dolutegravir-based first-line ART (database closure: 26 May 2023). VLs taken within 6 months of starting NNRTI- or dolutegravir-based ART were not considered. First-line ART consisted of either 2 nucleoside reverse transcriptase inhibitors (lamivudine combined with either tenofovir disoproxil fumarate, abacavir, or azidothymidine) and 1 NNRTI (efavirenz or nevirapine) or 2 nucleoside reverse transcriptase inhibitors and dolutegravir. For each core-agent class, we defined the first recorded measurement after at least 6 months on the respective regimen as baseline VL. Participants with virological failure (defined as a single VL ≥1000 copies/mL) at baseline were excluded. Participants were followed from the baseline VL of the respective ART core agent class until virological failure, change of ART core agent class, or last VL measurement.

VL tests of included participants were allocated to 2 groups. VL tests of participants taking NNRTI-based ART were allocated to the NNRTI group. VL tests of participants taking dolutegravir-based ART were allocated to the dolutegravir group. Participants who transitioned from NNRTI- to dolutegravir-based ART and met the inclusion criteria for both ART core agents contributed to both groups and had 2 different group-specific baseline VLs. In case of virological failure during follow-up, all subsequent VLs were censored, including those allocated to a different ART core agent group (ie, in case of contribution of VLs to both ART core-agent groups).

Within consecutive VL pairs, we assessed virological failure in the second VL (“outcome VL”) as the outcome of interest. The exposure variable was the immediately preceding VL (“exposure VL”), categorized into 3 groups: (1) viral suppression (<50 copies/mL); (2) low-range LLV (50–199 copies/mL); and (3) high-range LLV (200–999 copies/mL). Further variables included in the covariate analysis were demographic characteristics and history of ART (Table 1).

**Table 1.** Participant Characteristics

	NNRTI Group n = 13 043	Dolutegravir Group n = 13 604
Female, n (%)	8802 (67.5)	8825 (64.9)
Age in years at ART start, median (IQR)	37.8 (30.4–47.7)	37.8 (30.5–47.4)
Calendar year of ART start		
<2015	6958 (53.3)	5851 (43.0)
2015–2019	6085 (46.7)	6157 (45.3)
>2019	0 (0)	1596 (11.7)
CD4 count at diagnosis, n (%)		
<200 cells/µL	3447 (26.4)	2807 (20.6)
≥200 cells/µL	7589 (58.2)	6674 (49.1)
Missing	2007 (15.4)	4123 (30.3)
Age in years at baseline VL, median (IQR)^a^	41.6 (33.7–52.1)	44.3 (36.0–54.7)
Facility type at baseline VL, n (%)^a^		
Hospital	6885 (52.8)	5917 (43.5)
Health center	5925 (45.4)	7683 (56.5)
Missing	233 (1.8)	4 (<0.1)
Baseline VL, n (%)^a^		
Viral suppression	11 342 (87.0)	12 772 (93.9)
Low-range LLV^b^	1133 (8.7)	589 (4.3)
High-range LLV^b^	568 (4.3)	243 (1.8)
ART regimen at baseline VL, n (%)^a^		
TDF-3TC-EFV	10 112 (77.5)	NA
AZT-3TC-EFV	1376 (10.6)	NA
ABC-3TC-EFV	369 (2.8)	NA
TDF-3TC-NVP	508 (3.9)	NA
AZT-3TC-NVP	651 (5.0)	NA
ABC-3TC-NVP	27 (0.2)	NA
TDF-3TC-DTG	NA	13 033 (95.8)
AZT-3TC-DTG	NA	80 (0.6)
ABC-3TC-DTG	NA	491 (3.6)
VL pairs per participant, n (%)		
1	4693 (36.0)	7970 (58.6)
2	2676 (20.5)	4724 (34.7)
3	2312 (17.7)	719 (5.3)
4	1866 (14.3)	137 (1.0)
≥5	1496 (11.5)	54 (0.4)
Years of follow-up, median (IQR)	2.1 (1.1–3.2)	1.2 (1.0–2.0)

Abbreviations: 3TC, lamivudine; ABC, abacavir; ART, antiretroviral therapy; AZT, azidothymidine; IQR, interquartile range; LLV, low-level viremia; NNRTI, nonnucleoside reverse transcriptase inhibitor; TDF, tenofovir disoproxil fumarate; VL, viral load.

^a^First VL recorded after at least 6 months of therapy with the respective ART core-agent class.

^b^Low-range LLV: 50–199 copies/mL; high-range LLV: 200–999 copies/mL.

### Statistical Analysis

Descriptive statistics were summarized as frequencies and percentage for categorical data and medians with interquartile range (IQR) for continuous data. We derived incidence rates with 95% confidence intervals (CIs) of virological failure and LLV. For derivation of the LLV incidence rate, we did not distinguish between low- and high-range LLV and excluded participants with LLV at baseline. Within consecutive VL pairs, we assessed (1) the probability of virological failure in the outcome VL stratified by the exposure VL category and (2) the association of the exposure VL category with virological failure in the outcome VL using mixed-effect logistic regression models. For item 2, individuals and healthcare facilities were considered as nested random intercepts to adjust for facility clustering and multiple VL pairs at the individual level. Models were fitted separately on data from the NNRTI and dolutegravir group and adjusted for sex (female/male), age at time of exposure VL (<40/≥40 years of age), facility type where exposure VL was taken (hospital/health center or private clinic), and calendar year of ART initiation (<2015/2015–2019/>2019). The robustness of the results was assessed in several sensitivity analyses where we (1) excluded viral blips, defined as a VL in the low-range LLV category (50–199 copies/mL) preceded by viral suppression, or a VL in the low-range LLV category without a record of a preceding VL, (2) excluded participants with previous exposure to another core agent (NNRTI) from the dolutegravir group, (3) included participants with history of virological failure under NNRTI in the dolutegravir group, and (4) merged low- and high-range LLV into 1 LLV category (VL 50–999 copies/mL). All analyses were performed using Stata (StataCorp Version 16.1).

### Patient Consent Statement

The VICONEL cohort was approved by the National Health Research Ethics Committee of Lesotho in 2016 (ID134-2016), with yearly renewals since then (last renewal 16 May 2023, version ID134-2016-Renew 02). Consent is waived for the analysis of routine observational data.

## RESULTS

### Baseline Characteristics

**Figure 1.**
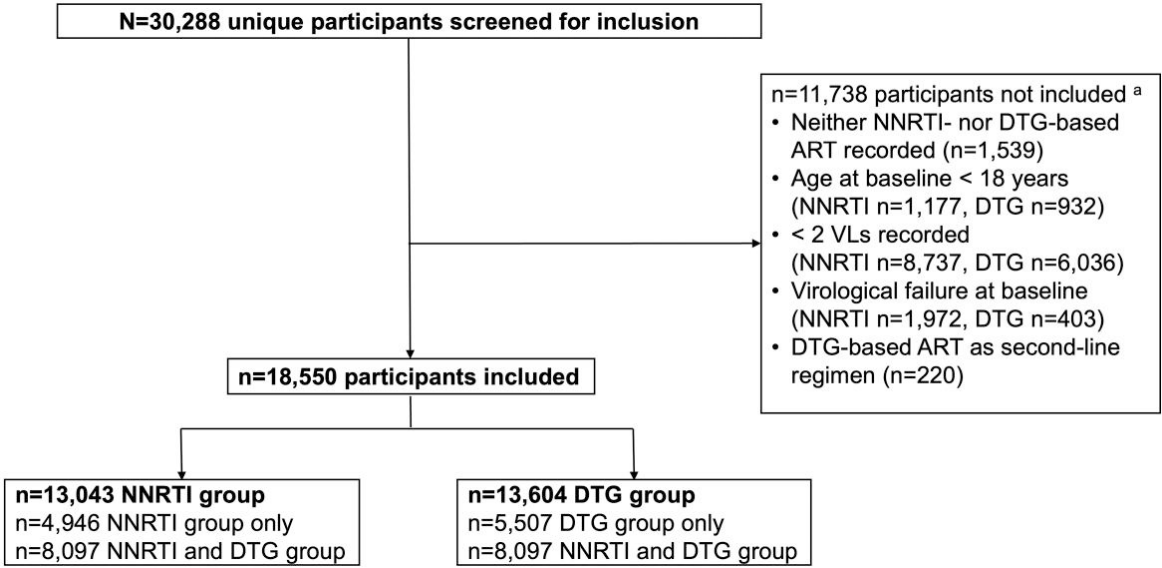
Study flow. VICONEL participants could contribute VLs to both the NNRTI and the DTG group in case of meeting the inclusion criteria for both ART core-agent classes. For each core-agent class, we defined the first recorded measurement after at least 6 months on the respective regimen as baseline VL. ^a^More than 1 criterion may apply. Abbreviations: ART, antiretroviral therapy; DTG, dolutegravir; NNRTI, nonnucleoside reverse transcriptase inhibitor; VICONEL, Viral Load Cohort North-East Lesotho; VL, viral load.

Among 30 288 VICONEL participants by 26 May 2023, 18 550 met the inclusion criteria (Figure 1). The characteristics of participants who were excluded are shown in Supplementary Table 1. Of the 18 550 included participants, 8097 (43.6%) were included in both the NNRTI and dolutegravir group, 4946 (26.7%) were included in the NNRTI group only, and 5507 (29.7%) were included in the dolutegravir group only. Compared with the dolutegravir group, participants in the NNRTI group were more likely to show virological failure at baseline (NNRTI group n = 1122/14 165 [7.9%] versus dolutegravir group n = 163/13 767 [1.2%]). Additonally including participants who experienced virological failure while taking NNRTI-based ART in the dolutegravir group, the proportion of participants with virological failure at baseline was 208/14 754 (1.4%) (Supplementary Table 2). Table 1 displays participant characteristics for the NNRTI and dolutegravir group separately. The majority of participants were female (NNRTI group: 67.5%; dolutegravir group: 64.9%). Median age at baseline was 41.6 years (IQR 33.7–52.1) and 44.3 years of age (IQR 36.0–54.7) in the NNRTI group and the dolutegravir group, respectively. In the NNRTI group, most participants were on an efavirenz-based regimen at baseline (11 857 [90.9%]).

### Incidence Rate and Occurrence of LLV and Virological Failure

In the NNRTI group, incidence rate of LLV was 9.5 events per 100 person-years (95% CI, 9.1–9.9) over a total person-time at risk of 23 631 years (Table 2). In the dolutegravir group, incidence rate of LLV was 3.4 events per 100 person-years (95% CI, 3.1–3.7) over a total person-time at risk of 17 790 years. Virological failure during follow-up occurred in 747 participants (5.7%; 95% CI, 5.3–6.1) with a median follow-up time of 2.1 years (IQR 1.1–3.2) in the NNRTI group and in 186 participants (1.4%; 95% CI, 1.2–1.6) with a median follow-up time of 1.2 years (IQR 1.0–2.0) in the dolutegravir group. In the NNRTI group, the incidence rate of virological failure per 100 person-years was 2.6 (95% CI, 2.4–2.8) with a total person-time at risk of 28 838 years. In the dolutegravir group, the incidence rate was 1.0 per 100 person-years (95% CI, .8–1.1) with a total person-time at risk of 19 253 years.

**Table 2.** LLV and Virological Failure

	NNRTI Group n = 13 043	Dolutegravir Group n = 13 604
Incidence rate per 100 person-years (95% CI)		
LLV^a^	9.5 (9.1–9.9)	3.4 (3.1–3.7)
Virological failure	2.6 (2.4–2.8)	1.0 (0.8–1.1)
Occurrence, % (95% CI)		
Virological failure	5.7 (5.3–6.1)	1.4 (1.2–1.6)
	747/13 043	186/13 604

	VL pairs within the NNRTI group n = 32 521	VL pairs within the dolutegravir group n = 20 411
VL category of exposure VL, n (%)		
Viral suppression	29 102 (89.5)	19 165 (93.9)
Low-range LLV^b^	2287 (7.0)	906 (4.4)
High-range LLV^b^	1132 (3.5)	340 (1.7)
Probability of virological failure, % (95% CI)	2.3 (2.1–2.5)	0.9 (0.8–1.1)
	747/32 521	186/20 411
Probability of virological failure, % (95% CI), stratified by exposure VL		
Viral suppression	2.0 (1.8–2.1)	0.8 (0.7–1.0)
	574/29 102	158/19 165
Low-range LLV^b^	3.9 (3.1–4.8)	1.8 (1.0–2.9)
	89/2287	16/906
High-range LLV^b^	7.4 (6.0–9.1)	3.5 (1.8–6.1)
	84/1132	12/340

Abbreviations: 95% CI, 95% confidence interval; LLV, low-level viremia; NNRTI, nonnucleoside reverse transcriptase inhibitor; VL, viral load.

^a^For the LLV incidence rate calculation, LLV was defined as a VL of 50–999 copies/mL. Participants with LLV at baseline were excluded from the model (included participants in NNRTI group: n = 11 342; dolutegravir group n = 12 772).

^b^Low-range LLV: 50–199 copies/mL; high-range LLV: 200–999 copies/mL.

In the NNRTI group, 13 043 participants contributed 32 521 VL pairs. Median time between the exposure VL and the outcome VL was 343 days (IQR 191–376). Participants contributed a median of 2 VL pairs (IQR 1.5–2.5). Among the 32 521 exposure VLs in the NNRTI group, 29 102 (89.5%) were virally suppressed, 2287 (7.0%) categorized as low-range LLV, and 1132 (3.5%) as high-range LLV (Table 2). Within VL pairs, the probability of virological failure in the outcome VL was 2.0% (95% CI, 1.8–2.5), 3.9% (95% CI, 3.1–4.%), and 7.4% (95% CI, 6.0–9.1) for viral suppression, low-range LLV, and high-range LLV in the exposure VL, respectively.

In the dolutegravir group, 13 604 participants contributed 20 411 VL pairs. Median time between exposure and outcome VL was 364 days (IQR 329–378). Participants contributed a median of 1.5 VL pairs (IQR 1.0–1.5). Among the 20 411 exposure VLs in the dolutegravir group, 19 165 (93.9%) were virally suppressed, 906 (4.4%) categorized as low-range LLV, and 340 (1.7%) as high-range LLV. Within VL pairs, probability virological failure in the outcome VL was 0.8% (95% CI, .7–1.0), 1.8% (95% CI, 1.0–2.9), and 3.5% (95% CI, 1.8–6.1) for viral suppression, low-range LLV, and high-range LLV in the exposure VL, respectively.

### Association of LLV With Subsequent Virological Failure

**Figure 2.**
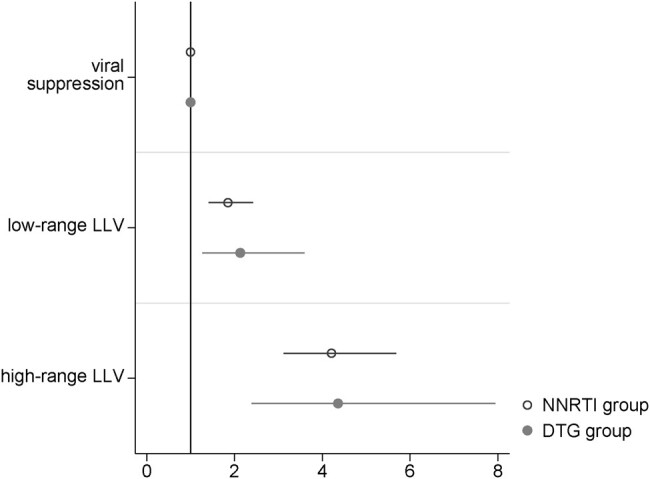
Association of the exposure VL category with virological failure in the outcome VL. Within VL pairs, a mixed-effects logistic regression model was used to assess the association of the exposure VL categorized as viral suppression (<50 copies/mL), low-range LLV (50–199 copies/mL), and high-range LLV (200–999 copies/mL) with virological failure in the outcome VL. Individuals and healthcare facilities were used as nested random intercepts to adjust for facility clustering and multiple VL pairs at the individual level. Models were fitted separately on data from the NNRTI and dolutegravir group and adjusted for sex (female/male), age at time of exposure VL (<40/≥40 y), facility type (hospital/health center), and calendar year of ART initiation (<2015/2015–2019/>2019). Abbreviations: 95% CI, 95% confidence interval; DTG, dolutegravir; LLV, low-level viremia; NNRTI: nonnucleoside reverse transcriptase inhibitor.

In the NNRTI group, compared with viral suppression, low-range LLV and high-range LLV in the exposure VL were both associated with virological failure in the outcome VL (adjusted odds ratio [aOR] 1.9; 95% CI, 1.4–2.4 for low-range LLV and aOR 4.2, 95% CI, 3.1–5.7 for high-range LLV; Figure 2 and Supplementary Table 3). In the dolutegravir group, compared with viral suppression, low-range LLV and high-range LLV in the exposure VL were likewise both associated with subsequent virological failure (aOR 2.1; 95% CI, 1.3–3.6 for low-range LLV; aOR 4.4; 95% CI, 2.4–7.9 for high-range LLV). Results remained consistent across the following sensitivity analyses: excluding viral blips in the low-range LLV category, excluding participants with previous exposure to NNRTI from the dolutegravir group, including participants with history of virological failure under NNRTI in the dolutegravir group, and defining LLV as ≥50 to <1000 copies/mL without distinction between low- and high-range LLV (Supplementary Tables 4–7).

## DISCUSSION

The 2021 WHO guidelines recommend clinical action on detection of any VL ≥50 copies/mL, rather than the previous threshold of VL ≥ 1000 copies. However, this change was based on data from the NNRTI era, and limited evidence currently exists on the association between LLV and subsequent treatment failure in the context of dolutegravir. In this study, we assessed the association of LLV with virological failure in people with HIV taking NNRTI- or dolutegravir-based first-line ART. Overall, unsurprisingly, occurrence of LLV or virological failure was less frequent in the dolutegravir group. However, both low- and high-range LLV were associated with subsequent virological failure in the case of either core agent. In participants taking dolutegravir, both low- and high-range LLV remained associated with subsequent virological failure across different sensitivity analyses, including those initiated on dolutegravir and those who experienced virological failure during a prior NNRTI-based regimen. Further, although the overall absolute probability of virological failure was lower among participants taking dolutegravir-based than among those taking NNRTI-based ART, the probability of virological failure remained higher for low-range and high-range LLV compared with viral suppression. These results indicate that the occurrence of LLV in people taking dolutegravir-based ART may have similar clinical implications as in NNRTI-based ART and should prompt healthcare providers to shorten the follow-up interval to next VL measurement and to investigate causes of LLV. Furthermore, these results imply that the revised WHO guidelines lowering the threshold of viral suppression based on evidence from studies conducted during the era of NNRTI-based ART may be transferred to the dolutegravir era.

Our study has several limitations. First, the NNRTI group had a longer follow-up time and a higher number of VL pairs per participant compared with the dolutegravir group because dolutegravir was rolled out in Lesotho starting in late 2019. Second, the VICONEL cohort collects only demographic and treatment metadata along with VL testing. Therefore, we were not able to assess adherence or other clinical or sociodemographic factors contributing to an unsuppressed VL. Third, similar to other cohorts in sub-Sahara Africa [20, 21], there was a considerable amount of loss to follow-up that was similar for both the NNRTI and dolutegravir group. Fourth, per national guidelines, VL tests are routinely done once per year only, making the differentiation between viral blips and LLV impossible. Therefore, we did not differentiate between viral blips and persistent LLV in the main analysis. Additionally, we defined virological failure as a single VL ≥ 1000 copies/mL because there was rarely a follow-up VL for confirmation within an appropriate time frame [19]. Fifth, we do not dispose of data about drug levels or drug resistance; therefore, we could not assess the impact of LLV on the emergence of drug resistance mutations and could not differentiate potential causes of LLV. Last, inherent to all cohort studies, there may be additional confounders we did not consider in our analysis.

To our knowledge, this is the first study assessing the association of LLV with virological failure in the context of dolutegravir-based ART in a resource-limited setting. Previously, a study from Nigeria used viral suppression in people taking NNRTI-based ART as a reference to report an association of LLV with virological failure for people taking dolutegravir-based ART [22]. Similar to our results, a previous study on people taking INSTIs in France reported an association of low-range LLV with virological failure defined as a VL > 200 copies/mL [23]. This definition differs from the one used in the latest WHO guidelines [1]. Another study assessing the association of LLV with virological failure in people taking dolutegravir-based ART was not able to report estimates because of limited events [24].

In our study, participants taking dolutegravir-based ART with low- or high-range LLV had higher odds of subsequent virological failure compared with viral suppression. This finding endorses close follow-up in any person with a VL ≥ 50 copies/mL, as now recommended in the 2021 consolidated guidelines of the WHO [1]. The recent scale-up of point-of-care VL platforms in remote areas across sub-Saharan Africa might, however, impose challenges because some assays have limited sensitivity at thresholds below 1000 copies/mL [1, 25].

For participants taking NNRTI-based ART, the incidence rate of LLV and occurrence of virological failure was similar to a large cohort study in South Africa [11]. Fewer data are available on incidence of LLV and virological failure on dolutegravir in limited-resource settings. Results from a multinational study in sub-Saharan Africa showed that the incidence rate of virological failure after transitioning to dolutegravir is 1.5 events per 100 person-years [26]. This incidence rate is in line with the results from our study, in which incidence rate of virological failure was 1.0 events per 100 person-years (Table 2). To our knowledge, there are no published studies on incidence of LLV occurring under dolutegravir in larger cohorts in resource-limited settings.

## CONCLUSION

The incidence of LLV and virological failure was lower in participants taking dolutegravir-based ART compared with NNRTI-based ART. However, LLV was associated with subsequent virological failure in the context of both NNRTI- and dolutegravir-based ART. This implies that LLV occurring in people taking dolutegravir may have similar clinical implications as for people taking NNRTI-based ART. Therefore, our data endorse the implementation of the revised 2021 WHO treatment monitoring guidelines recommending enhanced adherence counselling and close follow-up VL for people presenting with LLV—regardless of the core agent of their ART regimen.

## Supplementary Material

ofae013_Supplementary_Data

## Supplementary Data

Supplementary materials are available at *Open Forum Infectious Diseases* online. Consisting of data provided by the authors to benefit the reader, the posted materials are not copyedited and are the sole responsibility of the authors, so questions or comments should be addressed to the corresponding author.

## Notes

***Potential conflicts of interest*.** N. D. L. reports having received travel grants to attend scientific conferences from Gilead Sciences and ViiV Healthcare. He has received grants from SNSF, Fondation Botnar, Botnar Center for Child Health, the Swiss Agency for Development and Cooperation, and Moritz Straus Stiftung. His division received consultancy honoraria from ViiV Healthcare. J. A. B. and N. T. have received funds from the University of Basel Research Fund and the PANGEA Network. All other authors report no potential conflicts.
