# Supplementary material for: Clinical Relevance of Human Immunodeficiency Virus Low-level Viremia in the Dolutegravir era: Data From the Viral Load Cohort North-East Lesotho (VICONEL)
Source: Open Forum Infect Dis. 2024 Jan 8;11(2):ofae013. doi: 10.1093/ofid/ofae013 (PMC10883284; doi:10.1093/ofid/ofae013)
Supplement: ofae013_Supplementary_Data [file ofae013_supplementary_data.docx]

Supplementary material

- Supplementary table 1: characteristics of excluded participants
- Supplementary table 2: proportion of virological failure at baseline (dolutegravir)
- Supplementary table 3: association of the VL category of the exposure VL with virological failure in the outcome VL (main model)
- Supplementary table 4: sensitivity analysis excluding viral blips as exposure VLs
- Supplementary table 5: sensitivity analysis excluding participants with previous exposure to another ART core agent (NNRTI) from the dolutegravir group.
- Supplementary table 6: sensitivity analysis including participants in the dolutegravir group with virological failure during their prior ART core agent regimen (NNRTI)
- Supplementary table 7: sensitivity analysis with LLV defined as a single VL of 50-999 copies/mL

Supplementary table 1: characteristics of excluded participants

|  | | **n=11,738** |
| --- | --- | --- |
| Female, n (%) ^a^ | | 7,236 (63.0) |
| Age in years at ART start, median (IQR) ^b^ | | 32.1 (23.2-41.6) |
| Calendar year of ART start ^b^ | |  |
|  | < 2015 | 3,891 (36.2) |
|  | 2015-2019 | 4,623 (43.0) |
|  | > 2019 | 2,239 (20.8) |
| CD4 count at diagnosis, n (%) | |  |
|  | < 200 cells/µL | 1,774 (32.4) |
|  | ≥ 200 cells/µL | 3,708 (67.6) |

^a^ Data missing for n=247/11,738 (2.1%)

^b^ Data missing for n=985/11,738 (8.4%)

^c^ Data missing for n=6,256/11,738 (53.3%)

Abbreviations: ART, antiretroviral therapy, IQR, interquartile range

Supplementary table 2: proportion of virological failure at baseline VL (dolutegravir)

|  | **Dolutegravir group 1 ^a^**  **n=13,767** | **Dolutegravir group 2 ^b^**  **n=14,754** |
| --- | --- | --- |
| Proportion of virological failure at baseline VL, n (%) | 163 (1.2) | 208 (1.4) |

^a^ Group 1: application of all exclusion criteria except: virological failure at baseline

^b^ Group 2: application of all exclusion criteria except: virological failure at baseline; virological failure during prior NNRTI-based regimen

Abbreviations: VL: viral load

Supplementary Table 3: association of the VL category of the exposure VL with virological failure in the outcome VL (main model)

| **NNRTI group n=32,267** | | | **Adjusted OR** | **95% CI** |
| --- | --- | --- | --- | --- |
| VL category | | |  |  |
|  | | Viral suppression | Ref |  |
|  | | Low-range LLV | 1.9 | 1.4-2.4 |
|  | | High-range LLV | 4.2 | 3.1-5.7 |
| Sex | | |  |  |
|  | Male | | Ref |  |
|  | Female | | 0.7 | 0.6-0.9 |
| Age at exposure VL | | |  |  |
|  | | < 40 years of age | Ref |  |
|  | | ≥ 40 years of age | 0.8 | 0.7-1.0 |
| Facility type at exposure VL | | |  |  |
|  | | Hospital | Ref |  |
|  | | Health center | 0.9 | 0.6-1.3 |
| Calendar year of ART start | | |  |  |
|  | | <2015 | Ref |  |
|  | | 2015-2019 | 1.0 | 0.8-1.2 |
|  | | >2019 | no observations |  |
| **Dolutegravir group n=20,360** | | |  |  |
| VL category | | |  |  |
|  | | Viral suppression | Ref |  |
|  | | Low-range LLV | 2.1 | 1.3-3.6 |
|  | | High-range LLV | 4.4 | 2.4-7.9 |
| Sex | | |  |  |
|  | | Male | Ref |  |
|  | | Female | 0.7 | 0.5-0.9 |
| Age at exposure VL | | |  |  |
|  | | < 40 years of age | Ref |  |
|  | | ≥ 40 years of age | 0.5 | 0.4-0.7 |
| Facility type at exposure VL | | |  |  |
|  | | Hospital | Ref |  |
|  | | Health center | 1.0 | 0.8-1.4 |
| Calendar year of ART start | | |  |  |
|  | | <2015 | Ref |  |
|  | | 2015-2019 | 1.4 | 1.0-1.9 |
|  | | >2019 | 1.5 | 1.0-2.4 |

Abbreviations: NNRTI: non-nucleoside reverse transcriptase inhibitor; OR: odds ratio; 95% CI: 95% Confidence Interval; VL: viral load; LLV: low-level viremia, ART: antiretroviral therapy.

Supplementary Table 4: sensitivity analysis excluding viral blips as exposure VLs

| **NNRTI group n=30,207** | | **Adjusted OR** | **95% CI** |
| --- | --- | --- | --- |
| VL category | |  |  |
|  | Viral suppression | Ref |  |
|  | Low range LLV | 2.8 | 1.3-6.2 |
|  | High range LLV | 4.2 | 3.1-5.6 |
| **Dolutegravir group n=19,576** | |  |  |
| VL category | |  |  |
|  | Viral suppression | Ref |  |
|  | Low range LLV | 2.3 | 0.6-9.3 |
|  | High range LLV | 4.4 | 2.4-7.9 |

Definition of viral blip: (1) VL in the low-range LLV category (20-199 copies/mL) preceded by viral suppression or (2) VL in the low-range LLV category without preceding VL.

Abbreviations: NNRTI: non-nucleoside reverse transcriptase inhibitor; OR; odds ratio; 95% CI: 95% Confidence Interval; VL: viral load; LLV: low-level viremia.

Supplementary Table 5: sensitivity analysis excluding participants with previous exposure to another ART core agent (NNRTI) from the dolutegravir group.

| **n=8,320** | | **Adjusted OR** | **95% CI** |
| --- | --- | --- | --- |
| VL category | |  |  |
|  | Viral suppression | Ref |  |
|  | Low range LLV | 3.1 | 1.7-5.7 |
|  | High range LLV | 6.7 | 3.5-12.5 |

Abbreviations: OR; odds ratio; 95% CI: 95% Confidence Interval; VL: viral load; LLV: low-level viremia.

Supplementary table 6: sensitivity analysis including participants in the dolutegravir group with virological failure during their prior ART core agent regimen (NNRTI)

| **n=21,788** | | Adjusted OR | 95% CI |
| --- | --- | --- | --- |
| VL category | |  |  |
|  | Viral suppression | Ref |  |
|  | Low range LLV | 2.5 | 1.6-4.0 |
|  | High range LLV | 6.4 | 4.0-10.3 |

Abbreviations: OR; odds ratio; 95% CI: 95% Confidence Interval; VL: viral load; LLV: low-level viremia.

Supplementary table 7: sensitivity analysis with LLV defined as a single VL of 50-999 copies/mL

| **NNRTI group n=32,267** | | **Adjusted OR** | **95% CI** |
| --- | --- | --- | --- |
| VL category | |  |  |
|  | Viral suppression | Ref |  |
|  | LLV | 2.6 | 2.1-3.2 |
| **Dolutegravir group n=20,360** | |  |  |
| VL category | |  |  |
|  | Viral suppression | Ref |  |
|  | LLV | 2.7 | 1.8-4.1 |

Abbreviations: NNRTI: non-nucleoside reverse transcriptase inhibitor; OR; odds ratio; 95% CI: 95% Confidence Interval; VL: viral load; LLV: low-level viremia.
